# Amyloid Goiter Secondary to Behcet's Disease: A Case Report and Literature Review

**DOI:** 10.7759/cureus.49650

**Published:** 2023-11-29

**Authors:** Abdellatif Khader, Rajaa N Abed, Aysha R Rabee, Laith A Ayasa, Rose H Khrishi, Khaled A Judeh, Mohammad J Janazreh, Dima O Ibrahim

**Affiliations:** 1 General Surgery, Palestine Medical Complex, Ramallah, PSE; 2 Internal Medicine, Al-Quds University, Jerusalem, PSE

**Keywords:** thyroidectomy, hyperthyroidism, goiter, behcet disease, secondary amyloidosis

## Abstract

Amyloid deposition in the thyroid gland is a common presentation, yet amyloid goiter remains relatively rare. Proper differentiation of this condition from other goiter types and malignancies is essential. Although amyloid extensively invades the thyroid gland, patients are usually euthyroid, and many different presentations may occur. We report a case of a 42-year-old male patient who was diagnosed with secondary amyloidosis due to Behcet's disease. He presented with clinical manifestations of hyperthyroidism and systemic amyloidosis complicated by chronic kidney disease, which is the first case of such an entity to be reported in Palestine.

## Introduction

Amyloidosis is a clinical disorder in which amorphous, proteinaceous material is deposited in different organ tissues and alters their normal function. Amyloid deposition in large quantities in the thyroid gland can result in the formation of an enlarged gland known as amyloid goiter, which Beckman initially reported in 1858 and later Eiselberg in 1904 [1]. Both primary and secondary amyloidoses can cause amyloid deposition in the thyroid gland.

In patients with amyloid goiter, thyroid function tests are often non-specifically altered, and most patients are clinically euthyroid despite diffuse involvement of the disease [2]. Although deposition of amyloid protein in the thyroid gland is common, amyloid goiter is not an ordinary medical condition and is usually not diagnosed before surgery [3].

## Case presentation

A 42-year-old male patient presented to our hospital complaining of a minimally enlarged right thyroid gland since 2016. The patient has been a known case of Behcet's disease since 2005. It was complicated by secondary amyloidosis leading to nephrotic syndrome followed by chronic kidney failure, which eventually necessitated kidney transplantation in 2010.

Neck ultrasonography (US) revealed an enlarged, heterogenous thyroid gland without nodules. Fine needle aspiration (FNA) biopsy showed "in keeping with goitrous nodule, no evidence of malignancy." The patient's laboratory tests showed that he has subclinical hyperthyroidism, for which he was following up with a medical endocrinologist and taking thiamazole at a dosage of 40 mg per day. The patient was doing well until 2022 when he started to complain of a rapidly enlarging thyroid gland, from which he started to complain of dysphagia, dyspnea, and hoarseness.

On physical examination, a swelling was observed in the anterior aspect of the neck. Palpation revealed a bilateral, firm, non-tender, multinodular swelling that did not extend to the retrosternal area.

**Figure 1:**
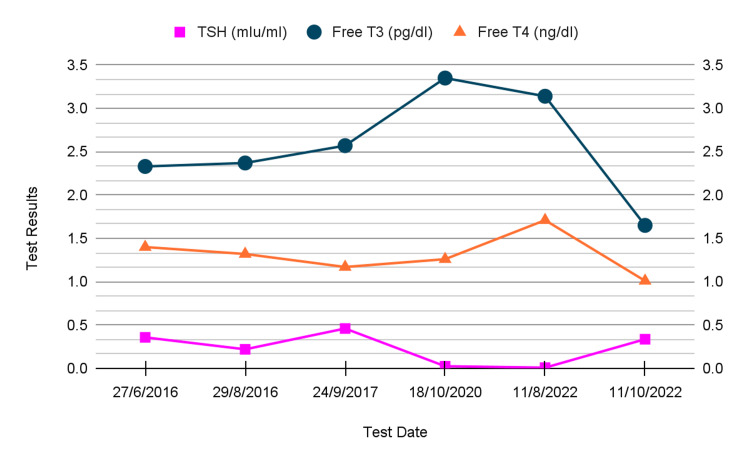
Thyroid function tests. TSH: thyroid-stimulating hormone; T3: triiodothyronine; T4: thyroxine.

Thyroid function tests, complete blood count values, and serum electrolytes were all non-remarkable except for phosphorus levels, which were minimally decreased, and kidney function tests were slightly elevated with a normal blood urine nitrogen (BUN) and a creatinine of 1.52 mg/dl (range 0.6-1.2 mg/dl). Figure 1 shows thyroid function test results during follow-up visits before surgery.

In August 2022, a neck computed tomography (CT) scan without contrast showed a markedly enlarged heterogeneous thyroid gland. Inferiorly, it extended to the level of the manubrium sterni, grossly encasing the common carotid arteries bilaterally with no definite invasion and also surrounding the trachea without compression or mass effect. Superiorly, it extended to the level of submandibular glands bilaterally. 

The neck US revealed bilateral enlargement of the thyroid lobes with a lobulated outline, extending to the thoracic inlet with a lateral deviation of the major neck vessels. No lymph node enlargement is observed.

Flexible fiberoptic laryngoscopy was performed and showed normal laryngeal anatomy, vocal cord movement, and no signs of any laryngeal pathology.

Subsequently, the patient underwent a total thyroidectomy; one of the parathyroid glands was accidentally removed and re-infiltrated within the left muscles of the neck. The laryngeal nerves were preserved. The postoperative course was uneventful, and the patient was discharged on the third postoperative day.

**Figure 2:**
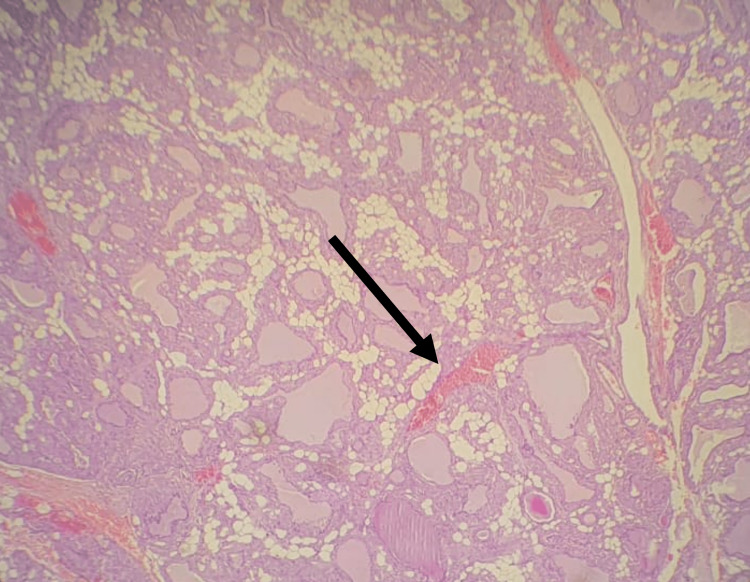
Deposition of amorphous, homogenous, eosinophilic substances in the thyroid tissue.

**Figure 3:**
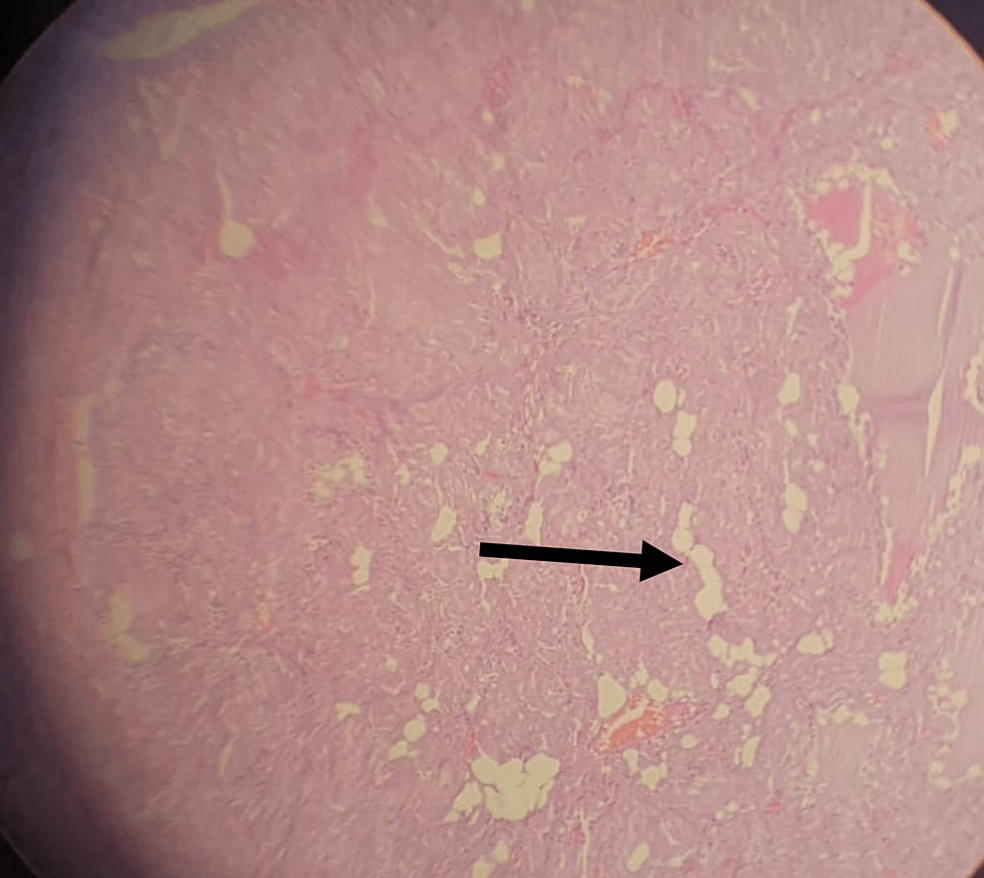
Fatty metaplasia of the thyroid gland.

Grossly, the thyroid gland appeared as grayish tissue measuring approximately 14 × 10 × 5 cm. Step sectioning revealed a fleshy cut surface. Histopathology revealed thyroid tissue exhibiting diffuse deposition of amorphous eosinophilic fibrillary material in the perifollicular and perivascular regions, which replaced the thyroid follicles in some areas (Figure 2), in addition to the presence of foci of fatty metaplasia (Figure 3).

## Discussion

Amyloidosis is a heterogeneous disease that results from the pathological deposition of insoluble amyloid proteins in different tissues, impairing their function. Amyloid is a beta-pleated sheet protein, which is originally a soluble protein that underwent conformational changes [4]. It accumulates in the extracellular matrix and creates a mass effect that impairs tissue function. Amyloid proteins can be deposited in many organs, each of which results in a different subtype of amyloidosis with different clinical presentations.

Amyloidosis is classified as primary (idiopathic cause) or secondary, resulting from a chronic inflammatory process (ankylosing spondylitis, rheumatoid arthritis (RA), familial Mediterranean fever (FMF), bronchiectasis) or from neoplastic disorders (medullary thyroid carcinoma), which are usually associated with amyloidotic renal failure [5]. 

Behcet’s disease is rarely complicated by secondary amyloidosis. According to reports, nine years is the typical period between the onset of Behcet's disease and amyloidosis, and eight years is the typical period between the onset of Behcet's disease and the detection of proteinuria [6]. Our patient had nephropathy within five years of the diagnosis of Behcet’s disease.

Amyloid goiter is the deposition of amyloid protein in the thyroid gland, resulting in significant rapid enlargement. It should be suspected in any case presenting with a euthyroid status associated with an enlarging diffuse goiter. Our patient was a known case of hyperthyroidism and had previously undergone FNA, which revealed a goitrous nodule with a minimal change in his thyroid function tests controlled by thiamazole. Our patient displayed an atypical clinical presentation of the disease given that he had a slowly enlarging thyroid gland since 2016, which then enlarged rapidly in a short period of time. 

This rapid enlargement of the thyroid in amyloid goiter can be attributed to several factors, including an acute increase in amyloid protein deposition within the thyroid gland, inflammatory responses associated with underlying systemic disease (Behcet's disease in the presented case), or a sudden change in hormonal balance. Additionally, secondary complications such as hemorrhage within the gland or superimposed infections could also contribute to rapid enlargement. While the exact mechanism in our patient's case is uncertain, these factors are likely contributors to the observed clinical presentation. The responsible mechanism for the diffuse proliferation of mature adipose tissue in the thyroid gland is still unclear.

The imaging modalities that are considered useful in investigating amyloid goiter may vary, as they include US, CT scans, and magnetic resonance imaging (MRI). FNA biopsy is a commonly used technique for evaluating thyroid lesions. In cases of suspected amyloid goiter, FNA biopsy can provide samples for histological examination. While FNA biopsy may not always provide a definitive diagnosis of amyloidosis due to sampling variability, it remains a useful initial investigative tool. In our case, the FNA biopsy played a crucial role in ruling out malignancy and guiding further diagnostic and treatment strategies. In cases of amyloid goiter, it may show an abundant irregular pink amorphous substance similar to those displayed in Figure 2. However, in the case of colloid amyloid, FNA will reveal solid hyaline-like substance. In amyloid goiter, approximately 10-40% of FNAs revealed only atypical follicular cells [7]. The precise diagnosis of amyloid goiter is obtained after histopathology. Histologically, amyloidosis is identified by the deposition of amyloid proteins, which can be specifically stained using Congo Red. Under polarized light, this stain exhibits a characteristic apple-green birefringence, which is considered a definitive feature for the presence of amyloid [7,8].

In our case, FNA was not adequate for the diagnosis. An amyloid goiter diagnosis is usually made postoperatively. If an amyloid goiter is diagnosed, the characteristics of amyloidosis should be identified.

It is important to highlight that surgery, specifically total thyroidectomy, is indicated not only for cosmetic purposes or when malignancy is suspected but also in instances of symptomatic relief from compressive symptoms such as dysphagia, dyspnea, and hoarseness. Furthermore, surgery may be considered when there is rapid gland enlargement suggesting malignancy, suspicion of hemorrhage within the gland, or when conservative management fails to provide symptom relief [8]. In our case, the indications for surgery extended beyond the cosmetic aspect to include the need for a definitive diagnosis and management of the patient's symptoms.

We conducted a computerized search to identify publications on amyloid goiter cases using PubMed from January 1, 2018 to February 2, 2023 (Table 1). The following keywords were used: amyloidosis, amyloid goiter, and secondary amyloidosis. The majority of patients had secondary causes of amyloidosis and complained of compressive symptoms (dysphagia, hoarseness, dyspnea). They were treated with total thyroidectomy, which pathologically relieved amyloid deposition. 

**Table 1:** Literature review of amyloid goiter. FNA: Fine needle aspiration; US: ultrasonography; M: male; F: female; RA: rheumatoid arthritis; UTI: urinary tract infection; FMF: familial Mediterranean fever.

Report	Age	Gender	Presentation	Cause of amyloidosis	Thyroid function test	US	FNA biopsy	Treatment	Pathology	Outcome
Patel Chavez et al. [9]	73	M	Dysphagia and hoarseness	Multiple myeloma	Euthyroid	Asymmetric goiter with three dominant nodules	Unsatisfactory	Total thyroidectomy and one gland parathyroidectomy	Benign thyroid parenchyma, diffuse amyloid deposition	Not available
López-Muñoz et al. [10]	48	F	Dysphagia	RA	Subclinical hyperthyroidism	Enlarged thyroid, isoechoic and without nodule	Amyloid deposition and adipose replacement of thyroid tissue	Total thyroidectomy	Diffuse adipose metaplasia of thyroid stroma, associated with adipose deposition of amyloid material in the vascular walls and thyroid interstitium	Uneventful
Kawai et al. [11]	60	F	Unconscious for six minutes, was dead	RA	Hyperthyroidism	Not done	Not done	-	Fat deposition, parenchyma destruction, and amyloid deposition. Amyloid deposits were also found in lung, pancreas, and kidney	Not available
Morado da Silva et al. [12]	54	F	Hoarseness and dysphagia	Papillary thyroid carcinoma	Euthyroid	Diffusely swollen thyroid and a well-defined nodule in the right lobe	Suspicious for follicular neoplasm	Total thyroidectomy	Papillary carcinoma and diffuse lipomatosis of the thyroid gland with amyloid deposition	Died after five months of diagnosis in secondary amyloidosis due to UTI complicated by sepsis
Jakubović-Čičkušić et al. [13]	40	M	Goiter, hoarseness, and breathing difficulty	Severe injury of right kidney and spine during war. Osteomyelitis. Two surgical interventions on both hips	Euthyroid	Enlarged thyroid with multiple cystic nodules in both lobes and presence of severe compression of surrounding neck structure	Non-diagnostic (thyroid biopsy showed amyloid deposition)	Total thyroidectomy	Fatty and fibrous tissue, with stains that showed amyloid deposition	After two years, patient is well on levothyroxine as a replacement therapy, no evidence of recurrence of the disease
Lari et al. [14]	53	M	Slowly enlarging thyroid gland during two years	Primary amyloidosis	Euthyroid	Enlarged thyroid gland with hyperechogenicity	Small amounts of adipose tissue with epithelial cells	Total thyroidectomy	Diffuse infiltration by mature adipose tissue, with attenuated scattered thyroid follicles, with extracellular amyloid deposition	Uneventful
Abukhalaf et al. [15]	23	M	Diarrhea, vomiting, cough, and chest tightness. During examination, enlarged neck mass was noted	FMF	Euthyroid	Enlargement of both thyroid lobes and isthmus, retrosternal thyroid extension, and no enlarged lymph nodes	Very suggestive of amyloid and negative of thyroid cancer	Patient recovered from adrenal crisis	Not done	Did not develop any significant or similar condition

## Conclusions

Amyloid goiter is a rare case, and a high index of suspicion is needed in patients with rapidly enlarging thyroid glands accompanied by a history of chronic inflammatory disease. FNA biopsy should be performed to exclude thyroid malignancy. A definitive diagnosis is made postoperatively following a histopathological examination. Every effort should be made to determine the extent of amyloidosis, and in those healthy patients, hematological malignancies should be ruled out.
